# Exploitation of alternative skin models from academia to industry: proposed functional categories to answer needs and regulation demands

**DOI:** 10.3389/fphys.2023.1215266

**Published:** 2023-06-02

**Authors:** Meital Portugal-Cohen, Dror Cohen, Ron Kohen, Miriam Oron

**Affiliations:** ^1^ DermAb.io, Haifa, Israel; ^2^ The Myers Skin Research Laboratory, Faculty of Medicine, School of Pharmacy, Institute for Drug Research, The Hebrew University of Jerusalem, Jerusalem, Israel

**Keywords:** alternative skin models, academia, industry, regulation, standardization, validation, data

## 1 Introduction

The utilization of alternative skin models (ASM) for testing has sparked significant attention in both academic and industrial environments. This aligns with the 3R principle, which stands for replacement, reduction, and refinement for ethical animal experimentation (Niehues et al., 2018; Sonawane et al., 2022).

In the field of dermatology, the development of ASM has become crucial (Mathes et al., 2014; Yun et al., 2018). Skin is a complex organ, and studying its biology and pathology often requires the use of animal models, which can be costly, time-consuming, react differently from human skin, and raise ethical concerns. ASM, based on human cells, reconstructed skin, *ex vivo* skin, organ-on-a-chip, and computational *in silico* models, have emerged as a promising solution, as they are more cost-effective and sustainable, present fewer ethical considerations, and, in certain cases, show advantages compared to the corresponding animal models (Danilenko et al., 2016).

The transformation of ASM from research to industry has been slow but steady, as summarized in Table 1 (Casas et al., 2013; Araújo et al., 2014; Mathes et al., 2014; Abaci et al., 2015; Hayden et al., 2015; Lukács et al., 2019; Wang et al., 2021; Mehling et al., 2022; Silva and Tamburic, 2022). The use of various complementary models can contribute to the industry by providing data for safety, claim substantiation, and initial proof of concept, diminishing technology readiness level (TRL) gaps and shortening the time to market. Furthermore, ASM will enable the study of underrepresented populations from diverse ethnicities and genetic backgrounds.

**TABLE 1:** Transformation timeline of ASM from research to industry.

Decade	ASM type	Functions and industrial sectors
1970s	Surgical waste or discarded skin	Developed for medical research to study skin diseases and drug and cosmetic effects (Araújo et al., 2014).
Early 1980s		As ethics-centered actions spearhead, the scientific community begins searching for alternative models to reduce animal experiments (Silva and Tamburic, 2022).
1980s and 1990s	3D-reconstructed skin model adoption	Cosmetic and personal care industry for safety and efficacy testing of new products, reducing the need for animal testing (Hayden et al., 2015).
Since early 2000s	*Ex vivo* skin models, such as skin explants, and starting skin-on-a-chip development	Pharmaceutical industry started using ASM for drug safety and efficacy testing, reducing reliance on animal testing and providing more accurate results (Mathes et al., 2014; Abaci et al., 2015).
Since early 2010s	3D-reconstructed skin models, *ex vivo* models	Medical device research and development, mainly for biocompatibility tests (Casas et al., 2013; Wang et al., 2021).
Late 2010s and early 2020s	Skin cells, 3D reconstructed skin models, skin-on-a-chip	• Additional industries, such as the chemical industry, have started to use ASM for toxicity testing, further reducing reliance on animal testing and improving accuracy (Mehling et al., 2022).
		• High-throughput model implementation, mainly in pharma and health-tech (Lukács et al., 2019).

Despite their importance and benefits, challenges and gaps still need to be addressed to facilitate the translation of ASM into industrial applications. One of the main needs is their standardization to ensure that results are consistent and comparable across different laboratories and models. Currently, the validation process for these models can be complex and expensive (Worth and Balls, 2001), which has been a barrier to their adoption. More research is needed to streamline the validation process and to ensure that ASM can replace traditional animal testing methods (Bas et al., 2021). Additionally, the lack of complexity of some models (e.g., cut off from blood vessels and immune cell migration) can limit their usefulness in certain applications. Finally, regulatory challenges also need to be addressed to ensure the safety and efficacy of ASM for use in different industrial sectors such as pharmaceuticals, devices, cosmetics, chemicals, and health-tech.

This opinion article discusses the needs, gaps, and challenges in adapting and using ASM for applied research to support the industry. Thus, it provokes the interplay among 1) the industrial fields with regulatory demands, 2) skin model categorization, 3) and the potential applications by using integrated data.

## 2 The different regulatory demands for each of the industrial fields

Development of alternative models for skin research has the potential to impact various industrial sectors, including cosmetics, pharmaceuticals, medical devices, chemicals, and health-tech. The challenge is to answer the needs, together with regulatory demands for each of the different industrial fields. The regulatory status of ASM varies depending on the industry and country. In general, regulatory agencies such as the U.S. Food and Drug Administration (FDA), European Medicines Agency (EMA), and the European Chemicals Agency (ECHA) encourage the use of alternative methods to animal testing and have developed guidelines for their validation and acceptance. The use of alternative methods is also supported by international organizations such as the Organization for Economic Co-operation and Development (OECD) and the International Council on Harmonization of Technical Requirements for Pharmaceuticals for Human Use (ICH). As a principle, safety and toxicity tests for new substances must rely on valid protocols. The European Centre for the Validation of Alternative Methods (ECVAM) has developed and validated several ASM, including reconstructed human skin models, which are widely used in the cosmetics industry, for example, OECD guidelines for testing chemicals for skin corrosion or irritation (OECD/OCDE 431/439) (Morales et al., 2016). In cosmetics, animal testing has been banned in the European Union and several other countries since 2013 (UNION, 2009). An increasing number of countries are lining up to follow this approach by the EU. Thus, non-animal skin models are widely applied as they can provide a cost-effective alternative to ensure product safety and efficacy either for raw materials like active ingredients or for the entire cosmetic formulations. There is an extensive range of efficacy tests and *in vitro*/*ex vivo* models for claim substantiation in cosmetics. Since these are not regulated, there is a high degree of freedom and creativity in selecting the models and assays (Götz et al., 2012; Zappelli et al., 2016; Filaire et al., 2022). The main claims supported by such efficacy models are regeneration, calming and soothing, brightening and firmness, and hydration (Tetali et al., 2020). It should be noted that in the cosmetic industry, ASM use is still not maximally exploited. This is mainly due to regulatory issues; for instance, the Asian market is quite demanding on this issue, accepting either only clinical studies or, in some cases, animal testing (Cheng et al., 2017). Additionally, the rather outdated definition of cosmetic products according to the EU guidelines (1223, 2009) can serve as an obstacle in the use of ASM or even for cell-based assays, particularly in non-competitive brands.

Despite the significant contributions of human skin models to the cosmetics industry, their potential benefits have not yet been fully realized in pharmaceutical research and development (Mathes et al., 2014). For drug development, these tissue models may be of particular interest for (Sonawane et al., 2022) systemically acting drugs applied on the skin; (Niehues et al., 2018) drugs acting at the site of application in the case of skin diseases or disorders. Delivery and distribution testing are crucial in the pharmaceutical industry. The FDA and the EMA require the use of alternative methods to animal testing wherever possible in line with the 3R principle. Furthermore, a new U.S. law has reduced animal testing requirements in some cases of drug development (économiques Odcedd, 2013). Medical devices are another sector that could benefit from ASM. *In vitro* and *ex vivo* models could be used to test the biocompatibility and efficacy of medical devices, such as wound dressings or transdermal drug delivery systems, before they are tested in animal models or human clinical trials. An additional need is to optimize device design and reduce the risk of adverse events in patients with regard to devices in contact with skin surface, such as patches, tapes, fabrics, and wearable devices. The FDA comes with the approach relying on certain parts of the Quality System Regulation (QS Regulation, 21 CFR 820) (Lincoln, 2012) that in specific wearable devices, such as those that are made from polymers, biocompatibility tests in animals can be omitted. Thus, ASM might be an appropriate replacement (Pellevoisin et al., 2018).

In the chemical industry, developing ASM could help address the growing concerns about the safety of chemical products for use in consumer products and industrial applications. Mainly, valid *in vitro* models of cells and reconstructed skin could be used to test the toxicity of chemicals, pesticides, and nano-particles and identify potential skin irritants, allergens, or sensitizers. The ECHA requires the use of alternative methods to animal testing for registration of chemicals under the Registration, Evaluation, Authorization, and Restriction of Chemicals (REACH) regulation (Fentem et al., 2021).

The health-tech industry has seen tremendous growth in recent years, with many innovations in various areas of healthcare and could also benefit from the development of ASM. For example, the database obtained from models that mimic the skin environment could be used to study skin diseases and develop new treatments, including personalized medicine approaches (Ingber, 2022). These models could also be used to develop skin substitutes for patients with severe burns or other skin injuries (Sierra-Sánchez et al., 2021).

## 3 Alternative skin model categorization

The ASM diversity reflects the need to cover safety and efficacy claims for different skin stressors and diseases and link them to different applications. Model categorization can be analyzed either from a traditional perspective based on model components and origin or from a new perspective suggested by authors, which is linked to functions and solutions for industrial needs.

The traditional perspective contains the following categories:

• Cells models: can be primary or cell-line models based on keratinocytes, fibroblasts, or melanocytes. They are appropriate for mechanism elucidation and certain factor isolation for initial proof of concept in dermatology (Ponec, 1992). Nevertheless, skin cell models are limited in their ability to replicate the complex structure and function of human skin, making it difficult to predict treatment behavior.
• 3D reconstructed skin models: attempt to mimic human skin’s structure and function by reconstructing human epidermis (RHE) or skin (RHS). They are created by combining different cell types, such as keratinocytes, fibroblasts, and melanocytes, in a three-dimensional structure; hence, different application types can be tested (Randall et al., 2018). However, 3D models do not perfectly mimic the complexity of human skin and their expensive production limits their accessibility.
• *Ex vivo* skin models: are used to study the effects of various treatments on human skin. Unlike reconstructed skin models, *ex vivo* models use real human skin tissue obtained from donors. Examples of *ex vivo* human skin models are skin explants, made by taking a small piece of skin tissue from a donor and maintaining it in culture (Eberlin et al., 2020), and skin organoid cultures, made by growing skin tissue in culture under conditions that mimic the *in vivo* environment, like basal cell carcinoma (BCC) and squamous cell carcinoma (SCC) (Lee and Koehler, 2021). Nevertheless, *ex vivo* model validation is not easy due to a high variation between donors. However, on the other hand, such variation exists in reality among people and should be considered. Perhaps, such nonuniformity is an advantage of *ex vivo* models and should be considered in validation.
• Skin-on-a-chip: is a type of microfluidic device consisting of a chamber that contains a layer of skin cells (e.g., keratinocytes, fibroblasts, and immune cells) that are cultured under conditions that mimic the *in vivo* environment. The chamber is connected to channels that allow for the flow of nutrients, oxygen, and other factors to capture the complex interactions between the skin and other organs or systems in the body, such as the immune system or the nervous system (Risueño Rojo et al., 2021). Yet, skin-on-a-chip models do not fully replicate the complexity of skin biology, and responses to stimuli may differ from those of real human skin.
• Skin *in silico* models: are part of the recent growing use of computational models allowing researchers to simulate and predict the behavior of skin under different conditions and develop new treatments. The challenge is to integrate, process, and analyze heterogenous data, which needs to be reliable and standardized (Filaire et al., 2022; dos Santos et al., 2020).

Our suggested perspective, as opposed to model catergorization, contains functionality categories, while each category might include different skin model types:

• Models imitating skin stressors and diseases: these models reflect skin conditions, disorders, and diseases (autoimmune, monogenic, etc.) for designated treatment development (Suhail et al., 2019). Part of these models has been used widely for years in both basic research and industrial sectors, e.g., wound healing (Greenhalgh, 2005), aging (Giacomoni and Rein, 2004), psoriasis (Bocheńska et al., 2017), atopic dermatitis (Eyerich et al., 2019), acne (Kanwar et al., 2018), and SCC (Yuspa, 1994), while part of them are less common, e.g., vitiligo (Boarder et al., 2021), alopecia (Žnidarič et al., 2021), and ichthyosis (Joosten et al., 2022). Additional models are based on co-cultures and skin inoculations by microorganisms for imitating infections, e.g., fungi for tinea pedis (Kim et al., 2019). These models can be further exploited in pharmaceuticals for future drug developments, as well as in cosmetics for coping with aging damages and cosmeceuticals for complementary treatment for skin diseases.
• Models for chemical safety and environmental protection: are valuable in testing skin reactions to exogenous elements, such as chemicals, radiation, and air pollution.

For chemical safety approval, models have to be valid and standardized. The safety of chemicals used in topical products such as cosmetics and personal care products is a growing concern. *In vitro* and *in silico* models have been developed to assess the safety of these chemicals before human testing (Viceconti et al., 2021).
  Models for environmental stressors can be used to prove protection efficacy by comparing damage markers before and after treatment (Farage et al., 2008; Portugal-Cohen et al., 2009). Ultraviolet (UV) radiation is a major contributor to skin damage, including premature aging, hyperpigmentation, and skin cancer (Portugal-Cohen et al., 2011). Studies investigating the effects of environmental pollution on the skin have been increasing in recent years. In particular, air pollution, through particulate matter (polycyclic aromatic hydrocarbons), and ozone, has been found to have detrimental effects on the skin (Portugal-Cohen et al., 2017).

• Models for modes of delivery: enabling examination of different forms of administration, such as topical, subcutaneous, and transdermal delivery. These kinds of models have to be characterized by the proper 3D structure and contain relevant skin layers, while cells models are not functional in this case. Trans-epidermal delivery can be performed by RHE. RHS and *ex vivo* explants are useful models for dermal and sub-cutaneous distributions (Vinken, 2020). The ratio of penetrated test material (drug, molecule, and formulation) is measured using analytical methods such as spectroscopy. For many years, Franz cells have been used mainly for skin penetration studies. Novel 3D models allow absorption and distribution studies of test materials in skin through methods such as fluorescence labeling (Pena et al., 2020).
• High-throughput platforms: these models might serve as sophisticated platforms for further research and applications in terms of new methodologies and valuable data. Examples of such models are skin microbiota, skin-on-a-chip by 3D printing, and models analyzed by omics (e.g., genomics, transcriptomics, proteomics, and metabolomics). Studying skin microbiota through alternative models is very challenging as skin microbiota is a complex and diverse community of microorganisms that play a critical role in maintaining skin health and protecting against harmful pathogens (Ron-Doitch et al., 2021a; Ron-Doitch et al., 2021b). Understanding the skin microbiota profile as a function of varying conditions can be a platform for novel treatment strategies for drugs and skincare products in the industry (Gueniche et al., 2022).

Skin-on-a-chip provides the infrastructure for complex interactions between the skin and other organs or systems (Tetali et al., 2020). Thus, different stressors can be induced and elucidated from this technology aspect and not only via other models such as reconstructed skin and *ex vivo* skin. Moreover, this model is an excellent tool for screening various drug-induced effects.
  Applying multi-omics analysis tools on different skin models provides another platform for valuable data and future development for personalized cosmetics and precision medicine (Theocharidis et al., 2022).

Although replacement of animal testing is a growing trend, it should be noted that animal use will not be replaceable in the foreseeable future, at least in the drug development field. This is because once a drug has a systemic effect, there are no good predictors in any of the available ASMs. However, the advanced organ-on-chip technology, which has been further combined into multi-organ chip interactions to mimic whole-body responses, can replicate the structure and function of human organs. This cutting-edge technology might give the future answer to the regulatory framework for assessing new therapeutic compounds (Van Norman, 2020).

## 4 Data analysis and processing

Reliable data is a demand from ASM adopted by the industry. The data can be exploited in different ways:

• Collecting data from different experiments, tests, and sites performed on the same models to increase repetitions and contribute to achieving more robust results.
• Gathering retrospective and prospective data from different ASM and comparing them to those of animal and clinical models can be a vital prediction tool for further effective treatment development.
• Integration of different skin biomarker levels and multi-omics data for finding hidden correlations to define specific models by the biomarker pattern.

The different ways to obtain data exploitation, together with computational models and bio-convergence, will result in a solid and dynamic database with the potential to drive significant progress to improve the health and well-being of people worldwide.

## 5 Discussion

ASM are tools bridging between academia and industry for many applications in the field of dermatology. Hence, we suggest a new aspect for their categorization according to their functionality and solutions for the different industrial sectors. In order to be exploited for industrial needs, academic research results are not sufficient as standalone, and the skin models should answer several conditions:

• Valid models or models that can be under validation in the future and demonstrate repeatability and standardization. Features such as donor age, ethnicity, gender, and other genetic background need to be defined for data classification when using *ex vivo* models and 3D reconstructed RHE and RHS. This can be performed by biomarker collection, omics analysis, and feature profiling and will significantly assist in treatment development for cosmetics or pharma based on the different population groups’ characteristics and responses to treatments, including unrepresented populations.
• Solid and integrated database for further analysis and prediction.
• Reliability in safety prediction versus animal studies. This is important as several cases of high numbers of false positive *in vitro* model results for new chemicals were described in the literature (Ates et al., 2016). Hence, new tools are developed: 1) *in silico* methods generating computational toxicological profiles using existing data to more accurately predict the toxicological profile of new ingredients; 2) data mining methods to identify more relevant protocols and end points for assays.
• Providing claim substantiation for efficacy.
• Cost-effectiveness allowing wide screening.
• Sustainability.

**FIGURE 1:**
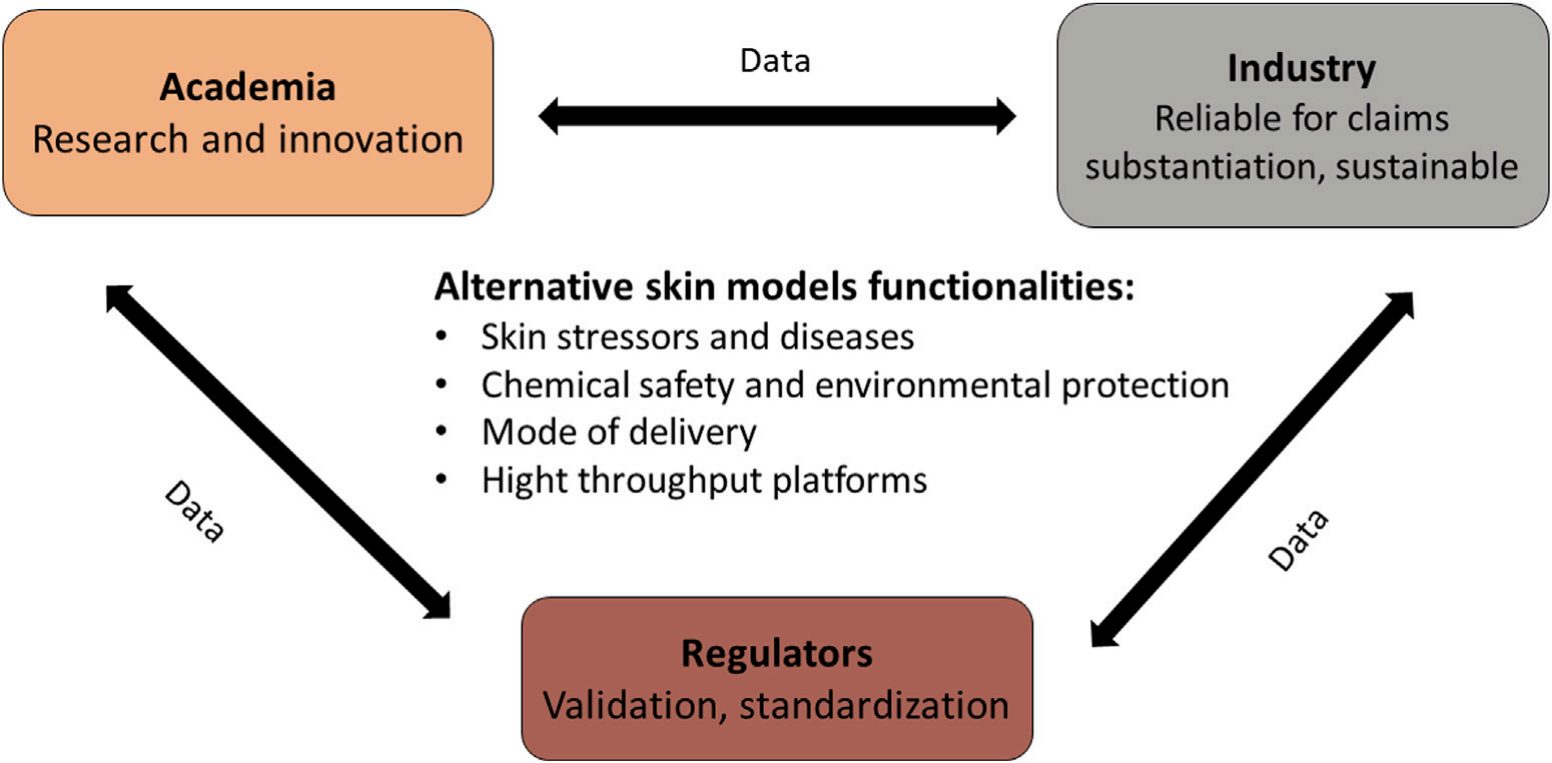
Alternative skin model needs and functionality.

In conclusion, ASM have the potential to revolutionize skin testing in both academic and industrial settings. Their use can significantly reduce the cost and time of product development and are more ethically and environmentally sustainable than animal models. Moreover, ASM can significantly reduce the number of volunteers in a potential clinical study, either for cosmeceutical or pharmaceutical research. However, several gaps still need to be addressed to facilitate their wider adoption in the industry, mainly standardization and validation. As summarized in Figure 1, addressing these challenges will require collaboration between all stakeholders: academia, industry, and regulatory bodies to ensure these skin models are valid and reliable.
